# CircPSMC3 alleviates the symptoms of PCOS by sponging miR‐296‐3p and regulating PTEN expression

**DOI:** 10.1111/jcmm.15747

**Published:** 2020-08-17

**Authors:** Jun Liu, Jinli Ding, Bing Qu, Jiuying Liu, Xiaojie Song, Qingli Suo, Aifen Zhou, Jing Yang

**Affiliations:** ^1^ Reproductive Medical Center Renmin Hospital Wuhan University Wuhan China; ^2^ Wuhan Children’s Hospital (Wuhan Maternal and Child Healthcare Hospital) Tongji Medical College Huazhong University of Science &Technology Wuhan China

**Keywords:** CircPSMC3, KGN cells, miR‐296‐3p, PCOS, PTEN

## Abstract

Polycystic ovary syndrome (PCOS), the most common female endocrine disease that causes anovulatory infertility, still lacks promising strategy for the accurate diagnosis and effective therapeutics of PCOS attributed to its unclear aetiology. In this study, we determined the abnormal reduction in circPSMC3 expression by comparing the ovarian tissue samples of PCOS patients and normal individuals. The symptom relief caused by up‐regulation of circPSMC3 in PCOS model mice suggested the potential for further study. In vitro functional experiments confirmed that circPSMC3 can inhibit cell proliferation and promote apoptosis by blocking the cell cycle in human‐like granular tumour cell lines. Mechanism study revealed that circPSMC3 may play its role through sponging miR‐296‐3p to regulate PTEN expression. Collectively, we preliminarily characterized the role and possible insights of circPSMC3/miR‐296‐3p/PTEN axis in the proliferation and apoptosis of KGN cells. We hope that this work provides some original and valuable information for the research of circRNAs in PCOS, not only to better understand the pathogenesis but also to help provide new clues for seeking for the future therapeutic target of PCOS.

## 1 INTRODUCTION

As the most common female endocrine diseases, polycystic ovary syndrome (PCOS) affects approximately 5−20% of women of reproductive age worldwide.^1^ The main characteristics of PCOS are chronic oligo‐anovulation and hyperandrogenemia, which are usually clinically manifested by irregular menstrual cycle, infertility, hairy and haemorrhoids.^2^ Not only that, PCOS also increases the risk of other diseases like obesity, hypertension, hyperlipidaemia and type 2 diabetes mellitus.^3^ Although chronic inflammation and oxidative stress have been linked to the pathogenesis of PCOS, the underlying molecular mechanism of PCOS remains unclear.^4^ Previous clinical studies showed that granulosa cells (GCs), which were closely related to the process of follicle formation and ovulation, exhibited aberrant cell death and proliferation in the ovaries of PCOS patients in contrast to normal ovaries.^5^ Recent evidence also indicated that suppressing the pathological changes in GCs can effectively alleviate PCOS symptoms.^6^ However, GCs are not adequate for the research of PCOS due to the difficulties in obtaining and maintaining the primary characteristics.^7^ Human granulosa‐like tumour cell line KGN, which retains the normal physiological features of GCs and overcomes the above problems, has been successfully employed to explore the precise functions and associated molecular mechanisms of GCs many times.^8^

Circular RNAs (circRNAs) are a type of non‐coding RNAs (ncRNAs) that lack canonical 5' cap and 3' poly A tail attributed to their covalent closed‐loop structures.^9^ As the competitive endogenous RNAs, circRNAs regulate the expression of multiple genes at the transcriptional and post‐transcriptional levels by sponging downstream microRNA(miRNA).^10^ Mounting evidences showed that the differential expressions of circRNAs were present in a variety of clinical illnesses such as cancers, CNS diseases, cardiovascular diseases, and some endocrine dysfunctions.^11^, ^12^, ^13^, ^14^ Recently, some reports aiming to probe into the differential expression profiles of circRNAs in GCs of PCOS patients found that 4 circRNAs were up‐regulated whereas 23 were down‐regulated.^15^ These circRNAs, which were abnormally expressed primarily in inflammation, proliferation and VEGF signalling pathways, indicated potential function in the occurrence and development of PCOS.

Although a series of differentially expressed circRNAs have been widely discovered in various diseases, little is known about their biogenesis processes and underlying mechanisms.^16^ It has been learned that the regulatory function of circRNAs mainly depends on its directly bound downstream miRNAs.^17^ MicroRNAs (miRNAs) are a type of conservative small regulatory non‐coding RNAs that can suppress the expression of target genes.^18^ As miRNAs are involved in key biological processes relied by cell life such as cell proliferation, apoptosis, development and differentiation, they are considered as vital regulators of gene expression.^19^, ^20^, ^21^, ^22^ Several reports have shown that miRNAs are mainly regulated by various lncRNAs and circRNAs to further exerting regulatory effects.^23^, ^24^ A previous research in mouse ovarian provides evidence that circEGFR could regulate the proliferation of GCs by sponging miR‐125a‐3p to regulate Fyn.^25^ It also reported that 3 circRNAs in cumulus cells of PCOS patients mediate the post‐transcriptional regulation of multiple genes by acting as sponges for miRNAs.^26^

In this study, we determined the abnormal reduction in circPSMC3 expression by comparing the ovarian tissue samples of PCOS patients and normal individuals. The symptom relief caused by up‐regulation of circPSMC3 in PCOS model mice suggested the potential for further study. In vitro functional experiments confirmed that circPSMC3 can inhibit cell proliferation and promote apoptosis by blocking the cell cycle in human‐like granular tumour cell lines. Mechanism study revealed that circPSMC3 may play its role through miR‐296‐3p/PTEN axis. We hope that our work contributes to the valuable insights into the circRNAs in the pathogenesis and treatment of PCOS by exploring the biological functions and molecular mechanisms involved in circPSMC3.

## 2 MATERIALS AND METHODS

### 2.1 Study approval

Ethical approvals were obtained from the Ethics Committee of the Renmin Hospital of Wuhan University. All collected samples were obtained subsequent to receive full written informed consent from the patients. Relevant animal experiments were conducted complied with the institutional ethical guidelines.

### 2.2 Cell lines and patient tissues

Human granulosa‐like tumour cell lines (KGN, COV434, SVOG) were acquired from the Suer Biological Technology (Shanghai, China). Cells were cultured in Dulbecco's modified Eagle's medium (DMEM, GE Healthcare Life Sciences, Logan, UT, USA) replenished with 10% foetal bovine serum (FBS, HyClone, South Logan, UT, USA) in a humidified incubator containing 5% CO_2_ at 37°C.

All samples were taken from women of reproductive aged who were 25 to 45 years old and did not receive any hormonotherapy at least 3 months prior to tissue sampling. PCOS patients were diagnosed based on the revised Rotterdam criteria provided by the American Society for Reproductive Medicine and the European Society for Human Reproduction and Embryology. Non‐PCOS women with regular menstrual cycles and normal ovarian morphology were identified as the control group. As described before, the human granulosa cells for subsequent RNA analyses were recovered and purified from the follicular fluid.^27^

### 2.3 Animal experiments

Sixteen 3‐week‐old female C57BL/6 mice were housed in our animal centre for 1 week after purchased from Shanghai Animal Centre. The PCOS mouse model was established by subcutaneous injection of 60 mg/kg dehydroepiandrosterone (DHEA, Aladdin, Shanghai, China) every day for 3‐6 weeks. Then, these mice were divided into two groups of 8 each, which received a subcapsular injection into the ovary of 5 × 10^8^ PFU/mL lentivirus carrying mock vector or circ‐PSMC3 vector (GeneChem, Shanghai, China). The ovarian tissues required for subsequent experiments were taken from mice killed after 2 weeks.

### 2.4 Serum insulin assay

After fasting the mice for 12 hours, 2 g/kg of glucose was intraperitoneally injected. Then, the orbital venous blood of above mice was extracted at 0, 30, 60, 90 and 120 minutes, respectively. Finally, the concentration of serum insulin was detected by mouse insulin ELISA kit (Westang, Shanghai, China), calculating the area under the curve (AUC) for further evaluation.

### 2.5 H&E staining

According to standard H&E staining protocol, mouse ovarian tissues were stained with haematoxylin and eosin solution (Beyotime, Shanghai, China) after fixed in formalin, paraffin‐embedded and sliced at 5 µm in turn. The images were captured and analysed using a fluorescence microscope (Olympus, Tokyo, Japan).

### 2.6 Cell transfection

Cell transfection is necessary to generate knockdown or overexpression model of circPSMC3 or miR‐296‐3p. SiRNA circPSMC3 (si‐circ), si‐negative control (si‐NC), circPSMC3 overexpression vector (circ‐PSMC3), blank vector (mock), miR‐296‐3p mimic, anti‐miR‐296‐3p, mimic NC and anti‐NC were all synthesized and obtained by Sangon Biotech (Shanghai, China). The sequences of si‐circ and si‐NC were as follows: 5'‐TAGGGTCCCTGCCCTTTGA‐3' and 5'‐UUCUCCGAACGUGUCACGUUU‐3'. The sequences were, respectively, transfected into cells with these siRNAs or plasmids by utilizing Lipofectamine 3000 reagent (Thermo Fisher, Waltham, Massachusetts, USA) according to manufacturer guidelines.

### 2.7 Quantitative reverse‐transcription‐polymerase chain reaction (qRT‐PCR)

The expression levels of circPSMC3, miR‐296‐3p and PTEN in the experimental groups were determined by qRT‐PCR. In strict accordance with the manufacturer's instructions, total RNAs were extracted by TRIzol reagent (Thermo Fisher Scientific, Massachusetts, USA). For circRNA, cDNA was reverse transcribed by a reverse transcription kit (QuantiTect® Reverse Transcription kit; Qiagen) and quantification was performed with a SYBR Green PCR Kit (SYBR® Green Realtime PCR Master Mix; Toyobo). For miRNA, QuantiTect® Reverse Transcription kit (Qiagen, German) was employed to reverse the total RNAs and SYBR Green PCR Kit (Toyobo, Japan) was used to examine the expression level of miR‐296‐3p. The qRT‐PCR primers were designed and synthesized by Sangon Biotech (Shanghai, China). The sequences of the primers used were listed as bellow: forward, 5′‐GTTTAGGGTCCCTGCCCTTTG‐3′ and reverse 5'‐ GTGTTGGGCTGGAAGCCATC‐3′ for circPSMC3; forward, 5'‐TGCCTAATTCAGAGGGTTGG‐3' and reverse, 5'‐CTCCACTCCTGGCACACAG‐3' for miR‐296‐3p; forward, 5'‐GTTTACCGGCAGCATCAAAT‐3 and reverse 5'‐CCCCCACTTTAGTGCACAGT‐3' for PTEN. Before calculation, we normalized the circRNA and miRNA expression levels by GAPDH or U6, respectively.

### 2.8 Western blot

KGN cells were lysed in RIPA lysis buffer (Sangon, Shanghai, China) to extract the total proteins, and the protein concentrations were evaluated by bicinchoninic acid (BCA) method (Beyotime Biotechnology, Shanghai, China). The 10% SDS‐PAGE (sodium dodecyl sulphate‐polyacrylamide gel electrophoresis) was employed to separate the extracted proteins, and separated proteins were transferred onto polyvinylidene fluoride membranes (Millipore, Schwalbach, USA). The membranes were blocked at 25°C for 1 hours by using 5% non‐fat dry milk powder, and then incubated at 4°C with primary antibody against PTEN (#3285, Cell Signaling and GAPDH (#ab181602, Abcam) overnight. Subsequently, the membranes were then conjunct with secondary antibodies (1:5000) at 25°C for 40 minutes. Finally, every protein band was visualized by enhanced chemiluminescence detection kit (Sangon Biotech, Shanghai, China) and related data were quantified by Image Lab Software.

### 2.9 Cell counting kit‐8 (CCK‐8) assay

The KGN cells were inoculated into 96 wells with the density of 4000 cells per well and incubated until cell attachment. Then, these seeded cells were cultured for 1, 2, 3, 4 and 5 days after transfection, respectively. Following treatment, 10 μL of the Cell Counting Kit‐8 (CCK‐8) solution (KeyGen, Nanjing, China) were supplied to every well. Finally, a microplate spectrophotometer (BioTek, VT, USA) was employed to detect the OD value of each well at 450 nmol\L.

### 2.10 EdU assay

The effects on the proliferation of KGN cells were measured by EdU assay using the BeyoClick™ EdU‐555 Kit (Beyotime Biotechnology, Shanghai, China) in accordance with manufacturer's protocols. Transfected KGN cells were seeded and cultured for 2 day. After fixed with 4% paraformaldehyde, cells were added with 5‐ethynyl‐2'‐deoxyuridine (EdU, 50 μmol\L each) and incubated at 37°C for 2 hours. These cells were subsequently stained with Apollo Dye Solution and DAPI. Finally, the EdU‐positive cells were visualized and analysed under the fluorescence microscope (Olympus, TKY, Japan).

### 2.11 Cell apoptosis assay

Cell apoptosis analysis was performed by using Annexin V‐Propidium Iodide (PI)/ fluorescein isothiocyanate (FITC) apoptosis kit (MSB, Hangzhou, China) and FACScan flow cytometer (BD Biosciences, NJ, USA). Briefly, the cell lines were stained with the fluorescent dyes FITC and PI for 20 minutes at 25℃ away from light. Subsequently, stained cells were washed and suspended with cold PBS for further flow cytometry studies within 1 hour.

### 2.12 Luciferase reporter assay

The pGL3‐basic vectors (GenePharma, Shanghai, China) with either circ‐PSMC3‐WT or circ‐PSMC3‐MUT were co‐transfected with miR‐296‐3p mimic or mimic NC into HEK 293T cells utilizing Lipofectamine 2000 reagent (Thermo Fisher). Correspondingly, the pGL3‐basic vectors with either PTEN‐WT or PTEN‐MUT were co‐transfected with miR‐296‐3p mimic or mimic NC into HEK 293T cells utilizing Lipofectamine 2000 reagent. The luciferase activities were measured by dual‐luciferase assay system after 48h.

### 2.13 RNA pull‐down assay

To pull down the miR‐296‐3p through circPSMC3, the cell lysates transfected with miR‐296‐3p mimics were incubated with biotin‐labelled probes of circ‐PSMC3‐WT or circ‐PSMC3‐MUT which were previously bound to magnetic beads. Similarly, to pull down the miR‐296‐3p through PTEN, the cell lysates transfected with miR‐296‐3p mimics were incubated with biotin‐labelled probes of PTEN‐WT or PTEN‐MUT which were previously bound to magnetic beads. After the pull‐down product was extracted, reverse transcription was carried out by qRT‐PCR.

### 2.14 Immunohistochemistry

After fixed in 4% paraformaldehyde, embedded in paraffin and sliced successively, the mouse model samples were incubated with the corresponding primary antibodies against PTEN (Catalogue#9552, Cell Signaling technology) at 4℃ for 24 hours. The images were captured and analysed using an optic microscope (Olympus, Tokyo, Japan).

### 2.15 Statistical analysis

The results of each experiment were repeated three times, and the experimental data were represented as means ± standard deviation (SD). The analyses were mainly interpreted using the software statistical SPSS version 19.0 (IBM, Chicago, USA). The statistical differences between control group and experimental group were calculated through the unpaired two‐tailed t test. Other statistical differences among multi‐groups were assessed by utilizing one‐way ANOVA or two‐way ANOVA. A *P *< 0.05 was determined statistically significant.

## 3 RESULTS

### 3.1 CircPSMC3 is markedly down‐regulated in PCOS patients and alleviates the symptoms in PCOS mice

**FIGURE 1:**
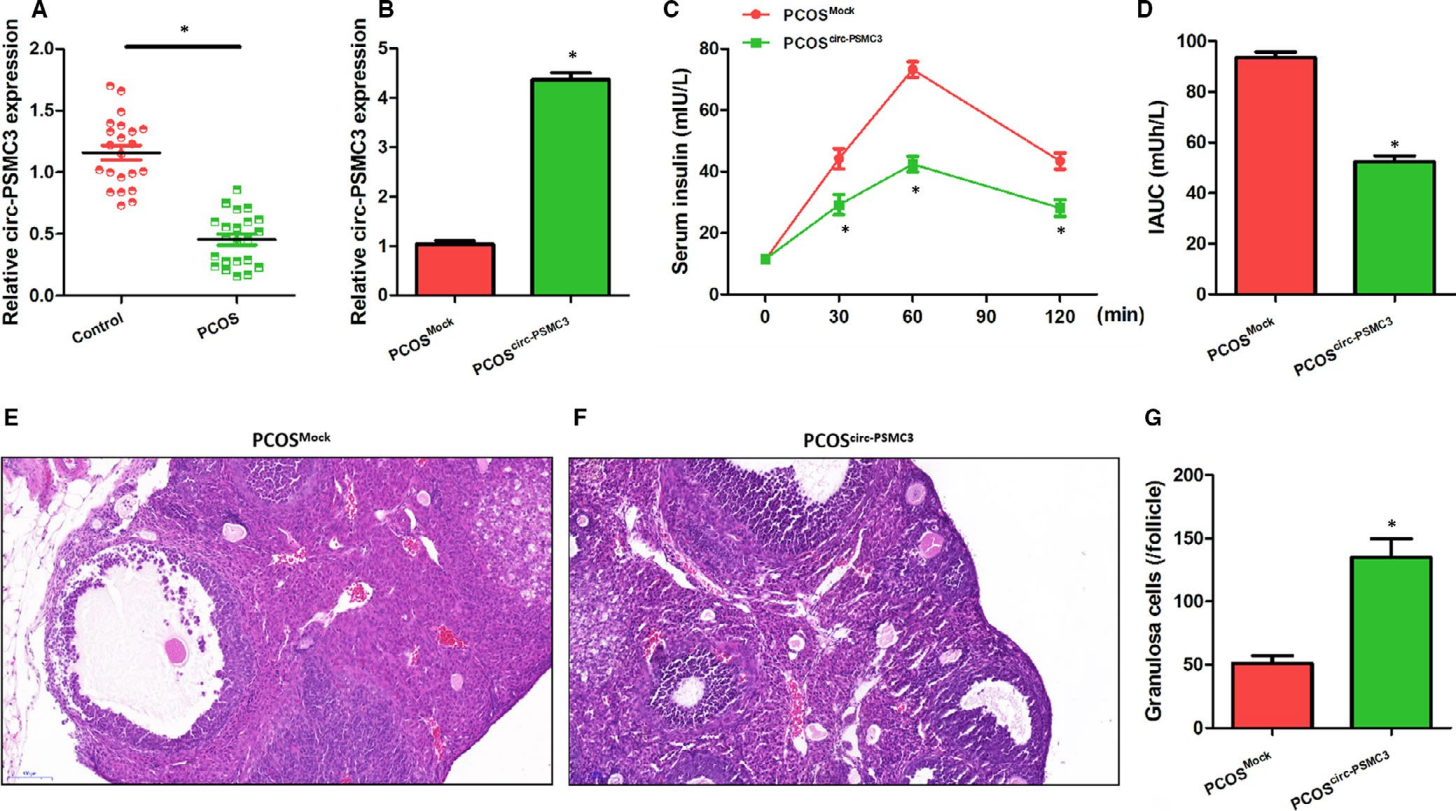
CircPSMC3 is markedly down‐regulated in PCOS patients and alleviates the symptoms in PCOS mice. (A) CircPSMC3 expressions were significantly down‐regulated in 22 PCOS patients compared with 22 normal individuals by using qRT‐PCR. (B) CircPSMC3 levels were increased in PCOS mice after circ‐PSMC3 lentivirus injection (n = 8 individuals per group). (C) Serum insulin levels were decreased in PCOS mice after circ‐PSMC3 lentivirus injection. (D) Area under the curve of serum insulin level (IAUC). (E, F) Representative H&E staining of ovary sections shows histopathological changes (Scale bar = 100 μm). (G) Quantification of the number of benign granular cells per follicle. N = 3 for each experiment, and the data were expressed as mean ± standard deviation (SD). *P < 0.05

To preliminarily explore the possible relevance between circPSMC3 and PCOS, we determined the mRNA levels of circPSMC3 in ovarian granulosa cells from 22 PCOS patients and 22 normal individuals through qRT‐PCR. The results showed that circPSMC3 expressed exceptionally low in PCOS specimens compared to the normal control group (Figure 1A). We overexpressed circPSMC3 in the PCOS mice model to further characterize the in vivo effect of circPSMC3 on this disease (Figure 1B). In the serum insulin release test, we found that circPSMC3 overexpression could effectively alleviate the increase in insulin release at different time points caused by sugar treatment (Figure 1C,D). Further analysing the H&E‐stained sections of ovarian tissues in these PCOS mice, the overexpression group showed a more regular tissue structure, a thicker granular cell layer and a larger number of benign granular cells compared with the control group (Figure 1E‐G). These results indicated that circPSMC3 might play a potential role in the biological process of PCOS, which deserves further research.

### 3.2 CircPSMC3 suppresses the viability and proliferation of KGN cells

**FIGURE 2:**
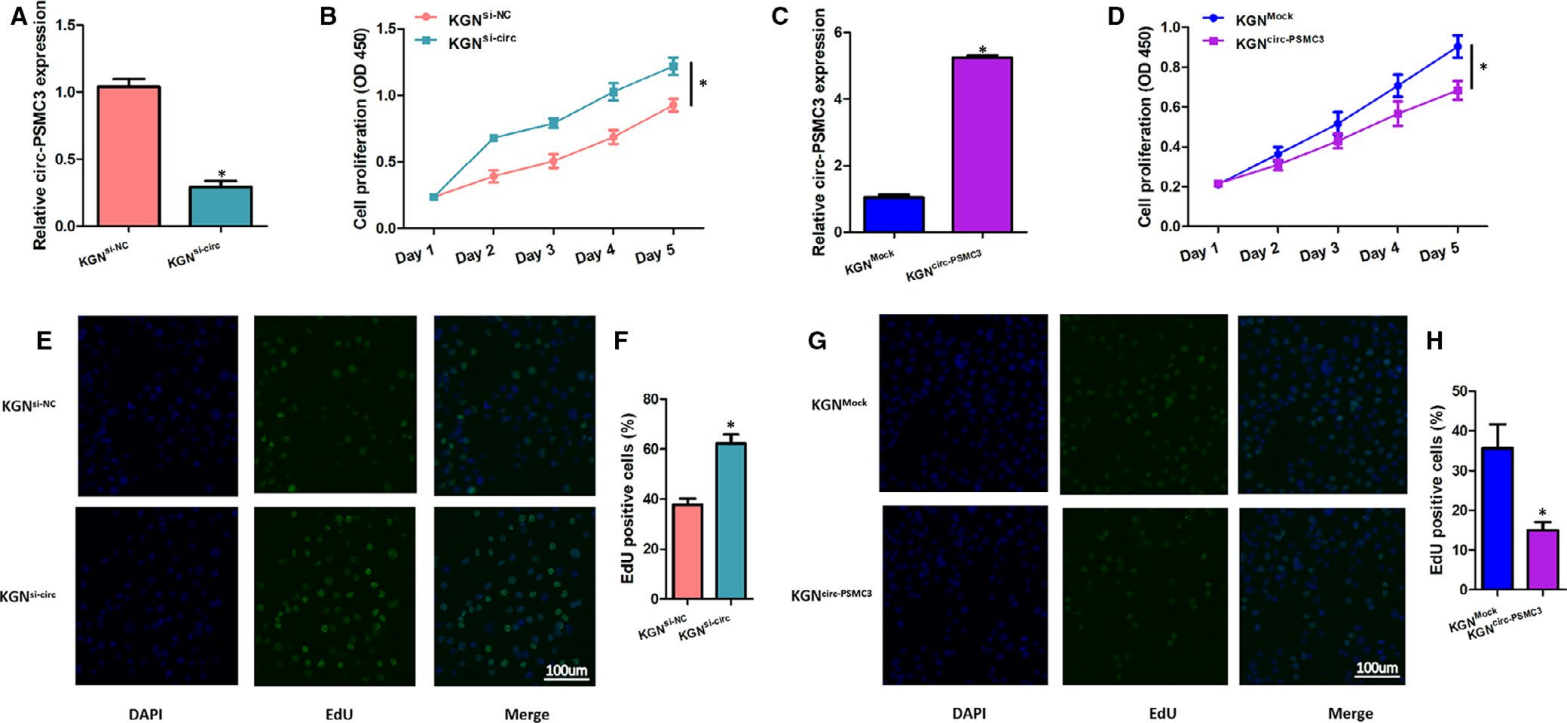
CircPSMC3 suppresses the viability and proliferation of KGN cells. (A, C) After transfection of cells with circPSMC3 vector or mock vector or si‐circ or si‐NC, qRT‐PCR was used to measure the expression of circPSMC3. (B, D) Cell growth curves were plotted with changes in absorbance over time in CCK‐8 assays (0, 1, 2, 3, 4 and 5 day). (E, G) Fluorescence images show EdU incorporated into nucleus. Blue and green indicate EdU and DAPI staining, respectively (Scale bar = 100 μm). (F, H) Percentage of EdU‐positive cells in KGN cells transfected with circPSMC3 vector or mock vector or si‐circ or si‐NC were performed to evaluate cell proliferation. N = 3 for each experiment, and the data were expressed as mean ± standard deviation (SD). *P < 0.05

The roles of circPSMC3 on viability and proliferation of KGN cells (human granulosa‐like tumour cell line) were determined. We designed si‐circPSMC3 (si‐circ) to decrease circPSMC3 expression level and circular transcript expression vector circPSMC3 (circ‐PSMC3) to overexpress circPSMC3 in KGN cells. After transfection, qRT‐PCR results were in line with expectations and showed a significant down‐regulation or up‐regulation of circPSMC3 levels in KGN cells (Figure 2A,C). The results of cell viability assay showed that circPSMC3 knockdown could significantly enhance the cell viability, whereas circPSMC3 overexpression might exert opposite role (Figure 2B,D). Besides, the EdU incorporation assay was adopted to measure cell proliferation. The results showed that si‐circPSMC3 transfection increased the number of EdU‐positive cells while circ‐PSMC3 transfection reduced this number (Figure 2E‐H). These above observations suggested that circPSMC3 inhibited the viability and proliferation of KGN cells.

### 3.3 CircPSMC3 promotes the apoptosis of KGN cells

**FIGURE 3:**
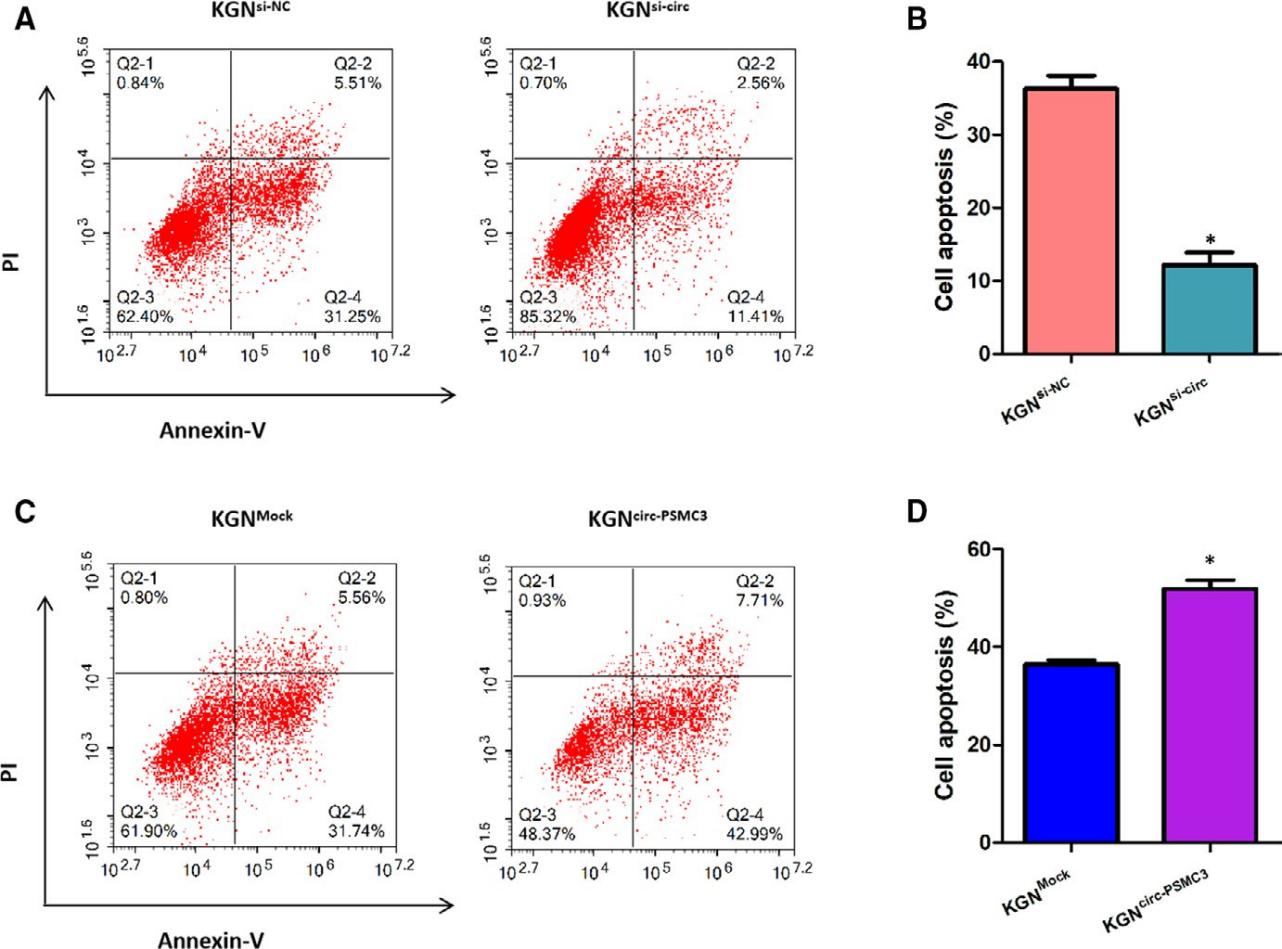
CircPSMC3 promotes the apoptosis of KGN cells. (A, B) The cell apoptosis ability was detected by flow cytometer using Annexin V/PI staining in KGN cells transfected with si‐circ or si‐NC. (C, D) The cell apoptosis ability was detected by flow cytometer using Annexin V/PI staining in KGN cells transfected with circPSMC3 vector or mock vector. N = 3 for each experiment, and the data were expressed as mean ± standard deviation (SD). *P < 0.05

The effects of circPSMC3 on apoptosis of KGN cells were identified. The cell apoptosis ability was analysed by flow cytometer using Annexin V/PI staining assay in KGN cells transfected with control or si‐circPSMC3 or circPSMC3 vector or Mock. Scatter plots presented that down‐regulation of circPSMC3 could decrease the amount of non‐viable apoptotic KGN cells whereas up‐regulation of circPSMC3 might increases the amount of non‐viable apoptotic KGN cells (Figure 3A,C). Stated in another way, there was a significant positive relevance between the ratio of apoptosis of KGN cells with the expression level of circPSMC3 (Figure 3B,D). The experimental results indicated that circPSMC3 promoted the apoptosis of KGN cells.

### 3.4 CircPSMC3 overexpression suppresses the cell proliferation and cell cycle progression of COV434 and SVOG cells in vitro

**FIGURE 4:**
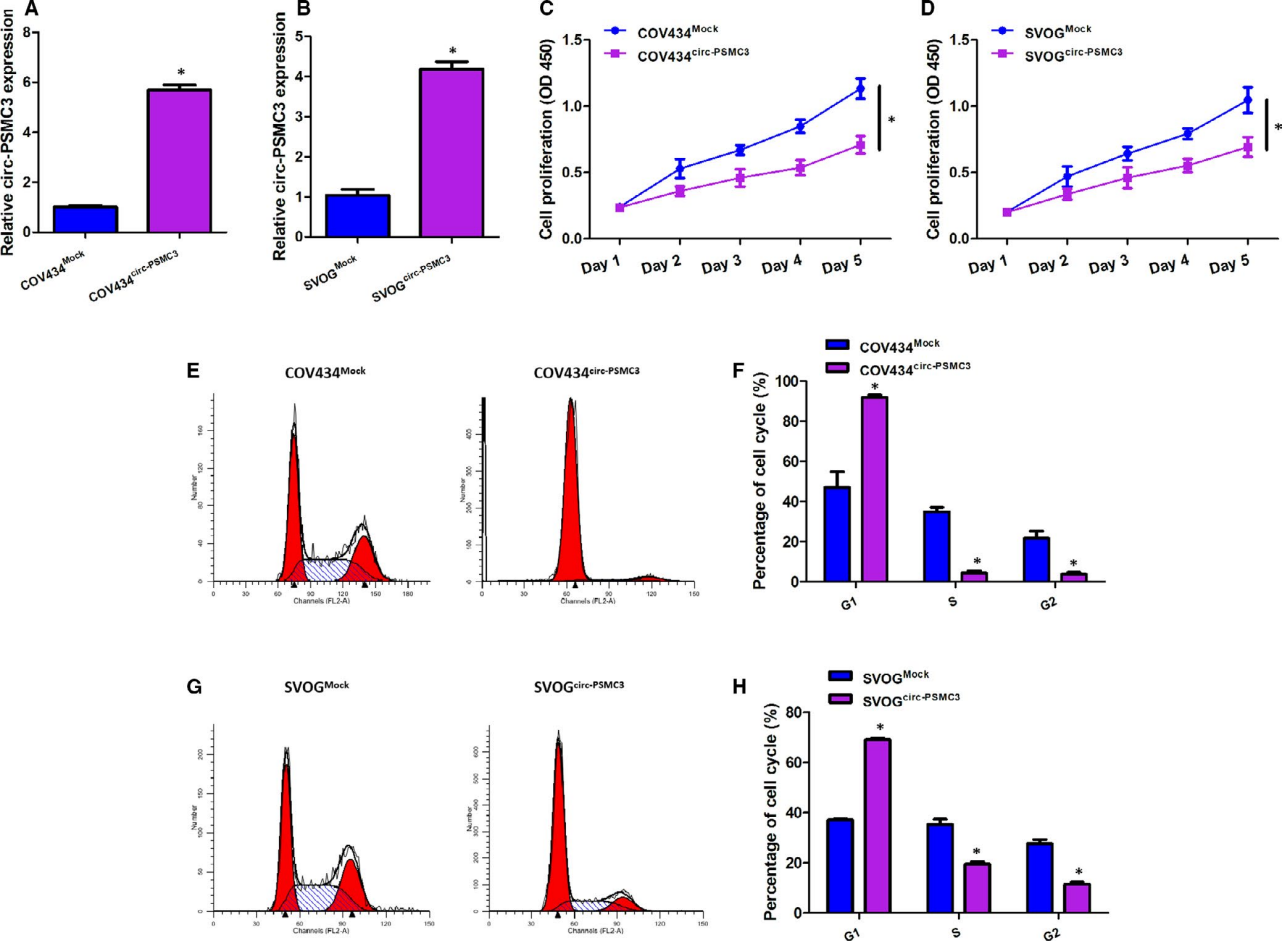
CircPSMC3 overexpression suppresses the cell proliferation and cell cycle progression of COV434 and SVOG cells in vitro. (A, B) CircPSMC3 expression was measured in COV434 cells or SVOG cells after transfected with circPSMC3 vector or mock vector. (C, D) Cell growth curves were plotted with changes in absorbance over time in CCK‐8 assays after transfected with circPSMC3 vector or mock vector in COV434 cells or SVOG cells (0, 1, 2, 3, 4 and 5 day). (E, G) Representative images of the cell cycle distribution of COV434 or SVOG cells. (F, H) Cell cycle analysis of COV434 or SVOG cells. N = 3 for each experiment, and the data were expressed as mean ± standard deviation (SD). *P < 0.05

Given the evidence of the KGN cell line alone is insufficient, we identified the effect of circPSMC3 on the cell proliferation and cell cycle in the other two human granulosa‐like tumour cell lines (COV434 and SVOG). CircPSMC3 is also highly expressed in the COV434 and SVOG cell lines (Figure S1A). We constructed the circPSMC3 overexpression models in these two cell lines (Figure 4A,B). Similar to the KGN cell line, circPSMC3 overexpression could also effectively suppress the proliferation of COV434 and SVOG cells (Figure 4C,D). Moreover, we analysed the difference in cell cycle distribution after up‐regulating circPSMC3 level by flow cytometry. We found that whether COV434 cells or SVOG cells, the percentage of cells in the G1 phase increased after circPSMC3 overexpression whereas the percentage of cells in the S and G2 phases decreased compared with the control (Figure 4E‐H). The above results suggested that circPSMC3 might affect the proliferation and apoptosis of granulosa‐like tumour cells through blocking the cell cycle progression.

### 3.5 Prediction and verification of the interactions between circPSMC3, miR‐296‐3p and PTEN

**FIGURE 5:**
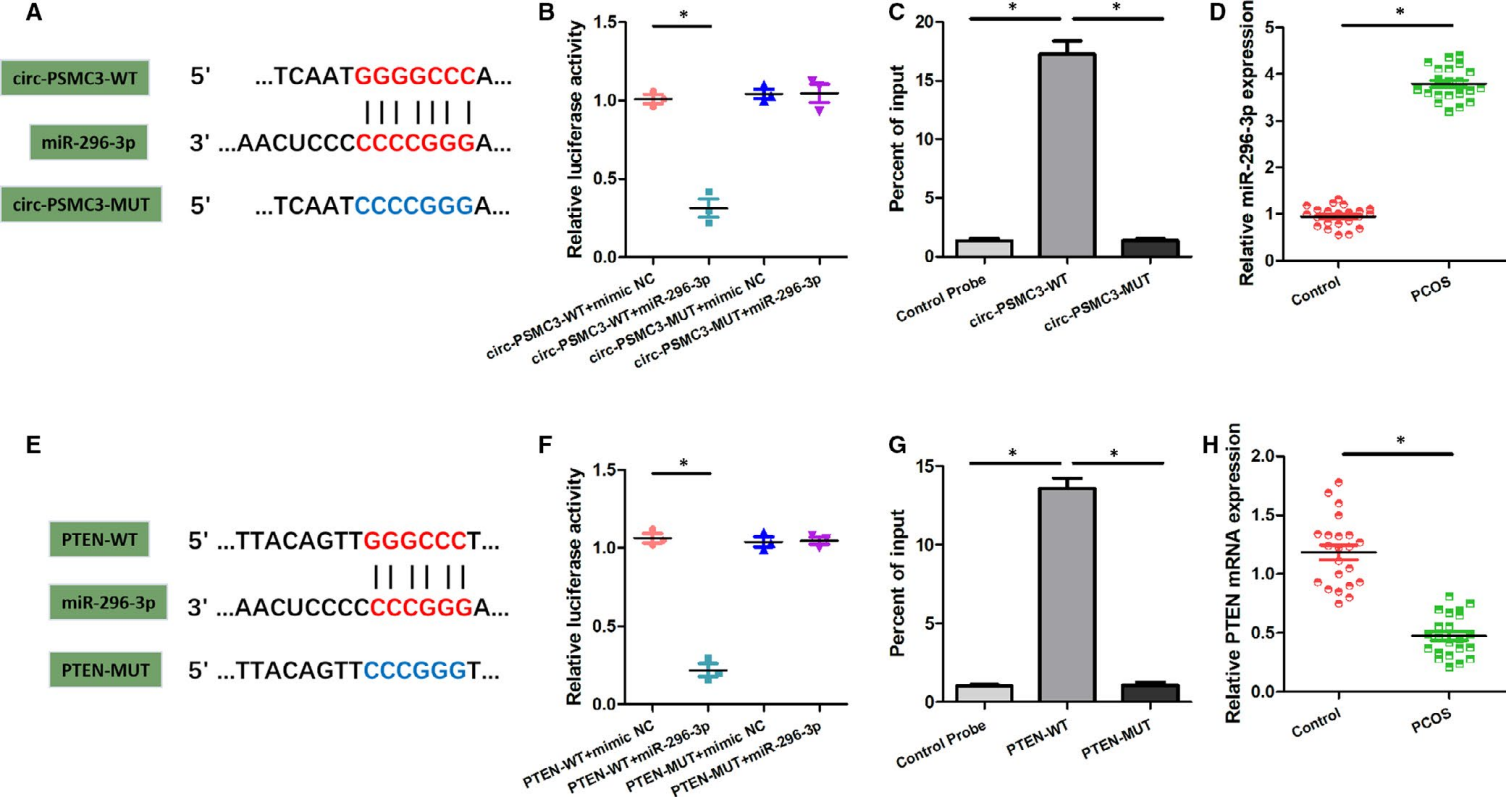
Prediction and verification of the interactions between circPSMC3, miR‐296‐3p and PTEN. (A, E) The inferred binding regions between circPSMC3 and miR‐296‐3p, PTEN and miR‐296‐3p. (B, F) Luciferase activities were detected in circPSMC3 WT and Mut or PTEN‐WT and MUT with miR‐296‐3p mimic or mimic NC co‐transfected cells. (C, G) RNA pull‐down assays were performed to determine the miR‐296‐3p enrichment on circPSMC3 WT and MUT or PTEN‐WT and MUT. (D, H) The relative expression of miR‐296‐3p and PTEN mRNA was measured in PCOS patients and normal individuals by using qRT‐PCR. N = 3 for each experiment, and the data were expressed as mean ± standard deviation (SD). *P < 0.05

Previous studies have reported that the realization of physiological function of circRNAs requires the presence of various binding miRNAs to regulate downstream proteins. In consequence, we resorted to the bioinformatic analysis (https://circinteractome.nia.nih.gov/index.html) to predict the direct binding target of circPSMC3. We found that the seed region of miR‐296‐3p has the sequence complementary to circPSMC3 (Figure 5A). In order to verify the website prediction, we conducted the luciferase activity assay and RNA pull‐down assay. The results showed that miR‐296‐3p could significantly reduce the luciferase activity of circ‐PSMC3‐WT‐transfected cells instead of MUT‐transfected cells (Figure 5B). In addition, the ability of circ‐PSMC3‐WT to pull down miR‐296‐3p was also offset by mutations (Figure 5C). Bioinformatic tools (http://www.targetscan.org/vert_72/) also forecasted Phosphatase and Tensin Homolog (PTEN) as a potential target for miR‐296‐3p (Figure 5E). Similarly, we confirmed the direct interaction between miR‐296‐3p and PTEN with the help of luciferase assay and RNA pull‐down assay (Figure 5F,G). Moreover, the differences in the expression levels of miR‐296‐3p and PTEN between PCOS patients and healthy control further confirmed the clinical relevance of this predicted axis (Figure 5D,H). Collectively, we forecasted and verified the physical basis of the circPSMC3/miR‐296‐3P/PTEN axis.

### 3.6 CircPSMC3 suppresses the proliferation of KGN cells by sponging miR‐296‐3p to regulate PTEN

**FIGURE 6:**
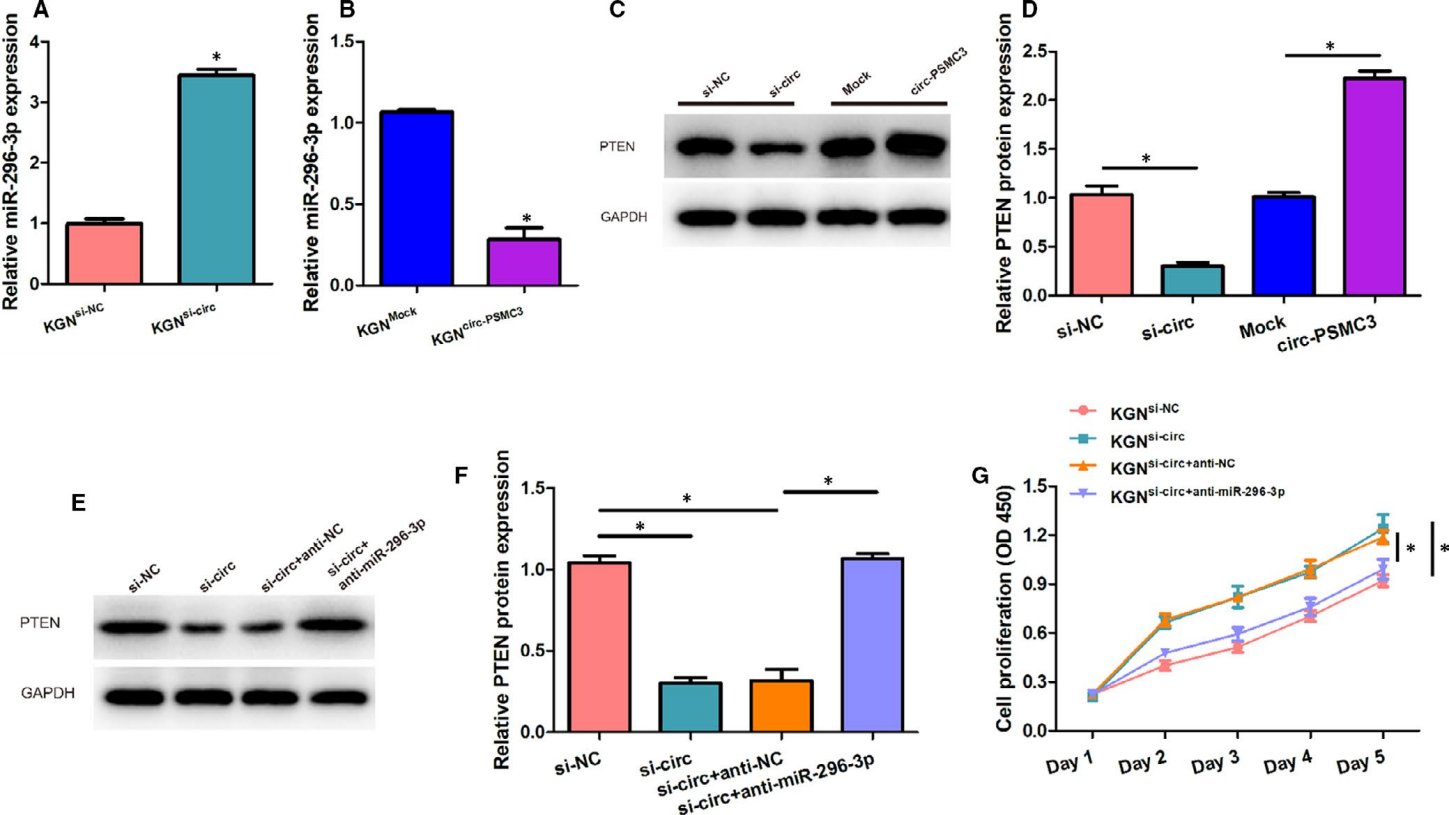
CircPSMC3 suppresses the proliferation of KGN cells by sponging miR‐296‐3p to regulate PTEN. (A, B) The expressions of miR‐296‐3p were detected in KGN cells transfected with circPSMC3 vector or mock vector or si‐circ or si‐NC through qRT‐PCR. (C, D) The expressions of PTEN protein were measured in KGN cells transfected with circPSMC3 vector or mock vector or si‐circ or si‐NC by Western blot. (E, F) The expressions of PTEN protein were measured in KGN cells transfected with si‐circ or si‐NC and anti‐miR‐296‐3p or anti‐NC through Western blot. (G) Cell growth curves were plotted with changes in absorbance over time in CCK‐8 assays after transfected with si‐circ or si‐NC and anti‐miR‐296‐3p or anti‐NC (0, 1, 2, 3, 4 and 5 day). N = 3 for each experiment, and the data were expressed as mean ± standard deviation (SD). *P < 0.05

We next determined the biological correlations and biological effects of the circPSMC3/miR‐296‐3P/PTEN axis in the KGN cells. QRT‐PCR results showed that circPSMC3 overexpression could cut down the miR‐296‐3p level in KGN cells whereas circPSMC3 knockdown had an opposite impact (Figure 6A,B). In addition, the Western blot assay hinted towards a positive correlation between circPSMC3 expression level and intracellular PTEN protein content (Figure 6C,D). To further confirm the key player of miR‐296‐3p in the regulation of circPSMC3 on PTEN, we co‐transfected si‐circ or si‐NC and anti‐miR‐296‐3p or anti‐NC into KGN cells. Western blot showed that anti‐miR‐296‐3p transfection eliminated the reduction in PTEN expression level induced by down‐regulating circPSMC3 (Figure 6E,F). Obviously, the regulation of circPSMC3 on PTEN expression was exerted through acting as a competitive endogenous RNA to sponge miR‐296‐3p. Additionally, we determined the effects of circPSMC3/miR‐296‐3P/PTEN axis on the proliferation of KGN cells. CircPSMC3 knockdown has been found to be beneficial for the proliferative capacity in our previous study, whereas down‐regulating miR‐296‐3p could dramatically offset this effect (Figure 6G). These facts indicated that circPSMC3 suppresses the proliferation of KGN cells by sponging miR‐296‐3p to regulate PTEN.

### 3.7 The relief of PCOS symptoms by circPSMC3 is achieved by up‐regulating the expression of PTEN in vivo

**FIGURE 7:**
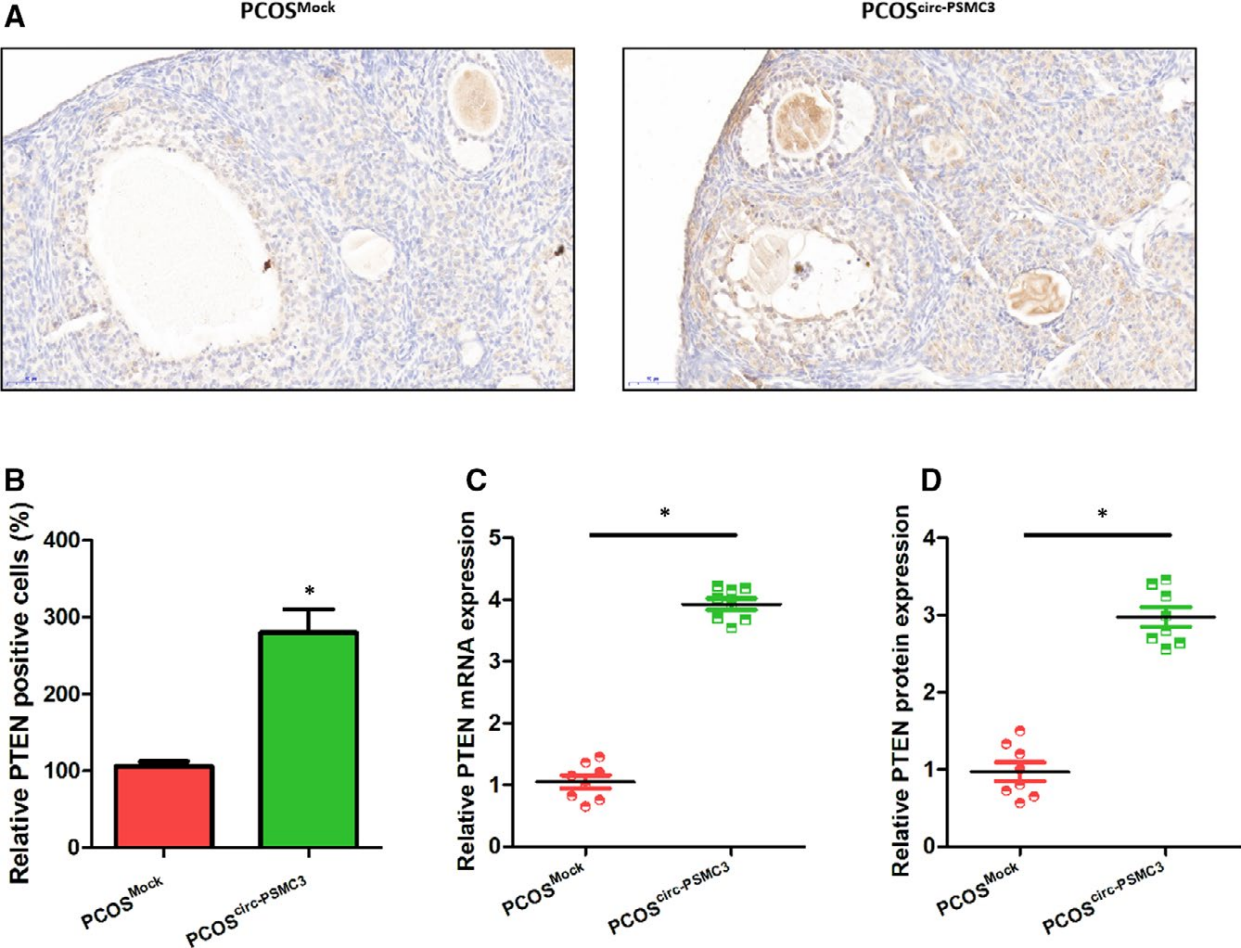
CircPSMC3 positively regulates PTEN expression in vivo. (A) Representative immunohistochemistry photomicrographs of PTEN expression in ovary sections (Scale bar = 100 μm). (B) Quantitation of PTEN‐positive cells. (C) The mRNA expressions of PTEN were significantly up‐regulated in PCOS mice after circ‐PSMC3 lentivirus injection (n = 8 individuals per group). (D) The protein expressions of PTEN were significantly up‐regulated in PCOS mice after circ‐PSMC3 lentivirus injection (n = 8 individuals per group). N = 3 for each experiment, and the data were expressed as mean ± standard deviation (SD). *P < 0.05

To further clarify the relationship between circPSMC3 and PTEN in the progression of PCOS, we quantified the PTEN levels in the ovarian tissues of model mice. We found that circPSMC3 overexpression greatly increased the number of PTEN‐positive cells (Figure 7A,B). Regardless of the PTEN mRNA level or protein level, 8 circPSMC3 overexpressing mice were significantly higher than the control group (Figure 7C,D). Obviously, the relief of PCOS symptoms by circPSMC3 is achieved by up‐regulating the expression of PTEN in vivo.

## 4 DISCUSSION

Serving as the major endocrine disease‐causing anovulatory infertility, PCOS plagues 5%‐20% of women of childbearing age in the whole world.^28^ However, searching the ideal biomarker for the accurate diagnosis and effective treatment of PCOS has been a formidable challenge because its pathogenesis has not been fully elucidated. Over the past decade, circRNAs were regarded as novel and vital regulators for the expression of genes as their altered expressions had been strongly associated with a variety of diseases.^29^, ^30^, ^31^, ^32^ Accumulating evidences suggest that circRNAs could be used as the excellent indicators of diagnosis and prognosis. For instance, the expression of circ_0074026 is significantly increased for patients with glioma, and this abnormal physiological phenomena indicates unfavourable prognosis.^33^ Circ_0001178 was observed to be highly expressed in colorectal cancer, which facilitated the process of metastasis and invasion of cancer cells in turn.^34^ Moreover, circ_0080425 expression in diabetic nephropathy tissues is significantly decreased and it markedly inhibits cell proliferation and fibrosis.^35^ Unfortunately, there is little research on the potential role and underlying molecular mechanism of circRNAs in the development of PCOS. Overexpression of circ_0023942 was reported to inhibit the proliferation of KGN and COV434 cells by regulating CDK4.^36^ Knockdown of hsa_circ_0118530 could reduce the damage of KGN cells by sponging miR‐136.^37^ However, in vitro cell line experiments alone cannot recapitulate the pathological condition of PCOS patients. In this study, we chose circPSMC3 as the starting point of our research and determined the differential expression level in clinical samples. Further in vivo and in vitro functional experiments showed that circPSMC3 can improve the symptoms of PCOS in mice model or human granulosa‐like tumour cell lines. With the help of bioinformatic tools, the potential bearers of the above functions are predicted and confirmed to be the circPSMC3/miR‐296‐3p/PTEN axis. To our knowledge, this is the first report to study the effect of circRNAs on PCOS in vivo.

CircPSMC3, a circular RNA derived from the PSMC3 gene, was originally discovered as a tumour‐suppressor factor for gastric cancer.^38^ Later studies found that circPSMC3 inhibited cell proliferation of nasopharyngeal carcinoma and prostate cancer by down‐regulating ROCK1 and DGCR8, respectively.^39^, ^40^ Recently, circPSMC3 was reported to suppress the invasion and migration of NSCLC cells by regulating the miR‐182‐5p/NME2 signalling pathway.^41^ Similar to most circRNAs, the major point of focus in circPSMC3 was also on tumour diseases. The disorder of cell proliferation and apoptosis is not only a patent for cancer, but also widely exists in other diseases, such as PCOS. As far as we know, our work is the only study to explore the role of circPSMC3 in non‐tumour diseases, which may provide some meaningful reference for the research of circPSMC3 in a wider range of diseases.

Whether it is miR‐296‐5p (miRNA‐296 from the 5' end) or miR‐296‐3p (miRNA‐296 from the 3' end), previous studies principally focused on their expression and role in human cancer. For instance, miR‐296‐3p was proved to increase tumour cell resistance to natural killer cells as an oncogene in prostate cancer, while exerted inhibitory effects on tumour cell migration and invasion in many types of malignancies such as choroidal melanoma, glioblastoma and non–small‐cell lung carcinoma.^42^, ^43^, ^44^, ^45^ Similar to its homolog, miR‐296‐5p has also been studied in the occurrence and development of various human cancers including oesophageal cancer, glioma and breast cancer.^46^, ^47^, ^48^ Except for miR‐296, many other miRNAs have been studied for their precise function and associated molecular mechanism in PCOS. MiR‐3940‐5p, miR‐140 and miR‐155 were found to promote GC proliferation while miR‐135a and miR‐30d‐5p induced apoptosis in GCs.^49^, ^50^, ^51^, ^52^, ^53^ We predicted and confirmed that miR‐296‐3p was involved in regulating the proliferation of KGN cells as a downstream target of circPSMC3. Three human‐like tumour cell lines used in this study were tested for miR‐296‐3p levels and found to have the lowest expression in SVOG cells (Figure S1B). Considering that most of the in vitro studies on PCOS used the KGN cells in the past, perhaps the SVOG cell line is a more appropriate choice.

PTEN, as a well‐known negative regulator of the PI3K/AKT pathway, has been studied as a tumour‐suppressor gene repeatedly.^54^ However, increasing evidence suggested that this signalling pathway is equally important for the initiation and progression of PCOS, which may affect insulin resistance and hyperandrogenism.^55^ Previous studies reported that miR‐17‐92 could promote cell proliferation and reduce the ratio of differentiated cells in bovine GCs by down‐regulating PTEN, suggesting that up‐regulating PTEN might be a strategy to suppress GCs.^56^ Consistent with it, our results showed that up‐regulating PTEN by circPSMC3 suppressed the proliferation of KGN cells. Nevertheless, what puzzles us is that miR‐200b and miR‐200c have been reported to reduce KGN cells proliferation by down‐regulating PTEN, which is the exact opposite of our conclusion.^57^ We speculate that some subtle changes have occurred in the regulatory pathway of PTEN in KGN cells compared with GCs. Clearly, this once again proved the limitations of the KGN cell line for studying the pathological mechanism of PCOS. Since KGN cells cannot fully reflect the true physiological characteristics of GCs, experiments with bovine GCs directly are obviously closer to the truth.

Our research still remains some limitations and weaknesses. Firstly, we used KGN cells instead of GCs in this study. As mentioned above, compared to the original GCs or SVOG cells, the KGN cell line does not seem to be the best choice for studying PCOS. We will search for the basis of the differences in the circPSMC/miR‐29‐3p/PTEN axis in these three cell lines in the follow‐up work. Additionally, since the focus of this work is on circRNAs, we have not studied the function and mechanism of miR‐296‐3p and PTEN in detail in vivo experiments. In the future, we plan to explore the potential functions and molecular mechanisms of these two in PCOS.

In conclusion, our finding reveals that circPSMC3 helps to alleviate the symptoms in PCOS mice via sponging miR‐296‐3p to up‐regulate PTEN expression level. We hope that our work contributes to the valuable insights into the better understanding of circRNAs in the occurrence and development of PCOS by exploring the biological functions and molecular mechanisms involved in circPSMC3.

## CONFLICT OF INTEREST

No potential conflict of interest was reported by the authors.

## AUTHORS' CONTRIBUTIONS

Jing Yang, Aifen Zhou and Jun Liu: Study design. Jun Liu, Jinli Ding, Bing Qu and Jiuying Liu: Experiments. Jun Liu, Xiaojie Song and Qingli Suo: Acquiring and collation of the data. Jing Yang, Aifen Zhou, Jun Liu and Jinli Ding: Writing the manuscript. All authors read and approved the final submitted manuscript.

## Supporting information

Fig S1

## Data Availability

The data used to support the findings of this study are available from the corresponding author upon reasonable request.
