# Supplementary figures and images for: CircPSMC3 alleviates the symptoms of PCOS by sponging miR‐296‐3p and regulating PTEN expression
Source: J Cell Mol Med. 2020 Aug 17;24(18):11001–11. doi: 10.1111/jcmm.15747 (PMC7521274; doi:10.1111/jcmm.15747)

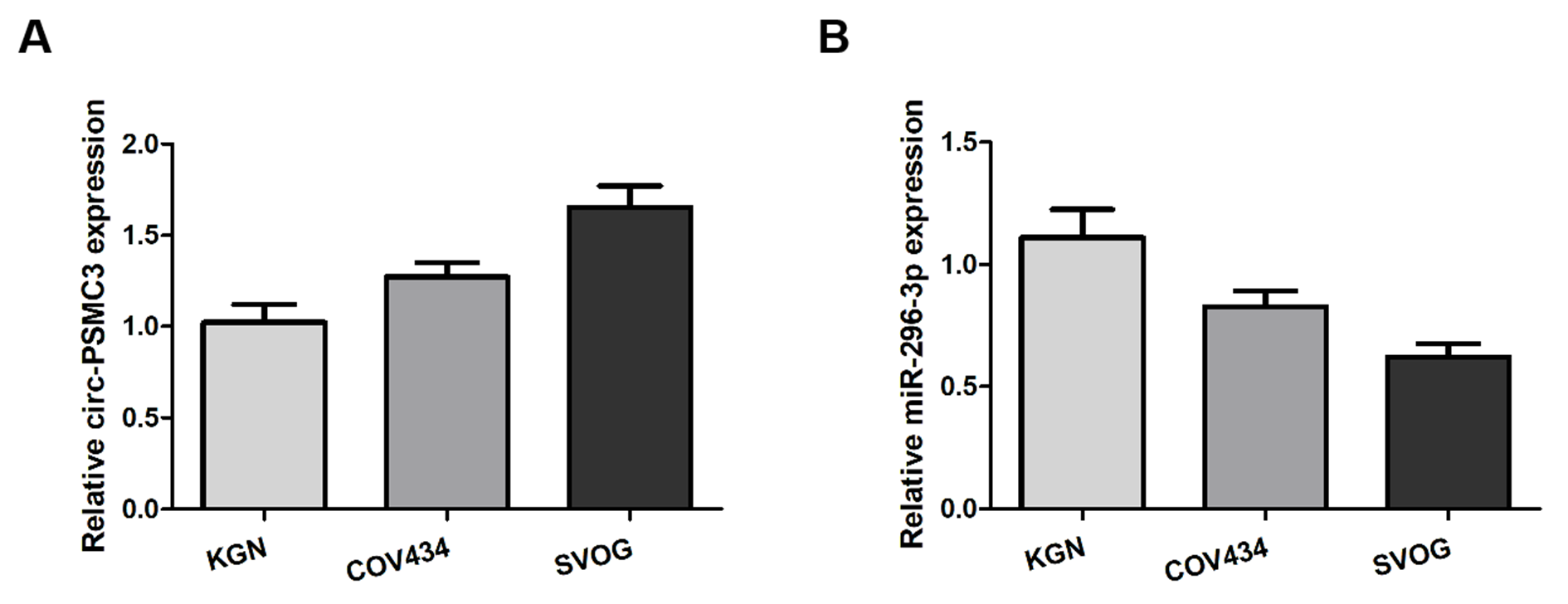

Supplement: Supplementary file 1 — Fig S1 [file JCMM-24-11001-s001.tif]
